# First reported case in Ireland of MEN2A due to a rare mutation in exon 8 of the *RET* oncogene

**DOI:** 10.1530/EDM-13-0044

**Published:** 2013-09-16

**Authors:** R Casey, S Prendeville, C Joyce, D O'Halloran

**Affiliations:** 1 Department of EndocrinologyCork University HospitalCorkIreland; 2 Department of PathologyCork University HospitalCorkIreland; 3 Department of BiochemistryCork University HospitalCorkIreland

## Abstract

We present the case of a 30-year-old female who was diagnosed with hereditary phaeochromocytoma secondary to a rare gene mutation in exon 8 of the *RET* oncogene. This genetic mutation was picked up as part of an extended genetic screen using a method known as next generation sequencing. Detection of this genetic mutation prompted further screening for the manifestation of multiple endocrine neoplasia type 2A (MEN2A). The patient subsequently underwent a thyroidectomy with histology confirming C-cell hyperplasia.

**Learning points:**

- Genetic analysis is an important step in the diagnostic work up of phaeochromocytoma.
- Extended genetic analysis is important when there is a strong suspicion of hereditary phaeochromocytoma.
- Mutations in exon 8 of the *RET* gene are associated with phaeochromocytoma as part of MEN2A syndrome.

## Background

Multiple endocrine neoplasia 2A (MEN2A) is a syndrome which predisposes to the development of medullary thyroid carcinoma, unilateral or bilateral phaeochromocytoma and primary hyperparathyroidism. Most cases of MEN2A occur due to mutations that affect codons on either exon 10 (codons 609, 611, 618, 620) or exon 11 (codon 634) of the *RET* gene. Familial medullary thyroid cancer (FMTC) has been linked to mutations in exon 8 of the *RET* gene, but the association with phaeochromocytoma and MEN2A has only recently been discovered.

The role of genotyping in the diagnosis and management of phaeochromocytoma is rapidly expanding. Recognition of the MEN2A phenotype in association with a mutation in exon 8 is extremely important as it ensures that patients with apparently sporadic phaeochromocytoma are followed-up and screened for C cell hyperplasia and medullary thyroid cancer. As the relationship between exon 8 mutations of the *RET* gene and familial phaeochromocytoma has only recently been discovered, it highlights the importance of extended genetic analysis in cases of apparently sporadic phaeochromocytoma in young patients or those with a strong family history.

Reporting of cases with this rare genetic mutation is essential in not only raising awareness amongst physicians about the importance of genotyping in phaeochromocytoma but also ensuring that genotyping is performed in dedicated laboratories where extended genetic sequencing is carried out to rule out false-negative reports.

## Case presentation

A 30-year-old female originally from Slovenia was first noted to have asymptomatic hypertension in 2008 during a routine check-up at a gym. She was referred initially to a general physician who performed a 24-h blood pressure monitor, which revealed a mean systolic pressure of 160 mmHg and a mean diastolic pressure of 110 mmHg. A work up for a secondary cause of hypertension included a renal ultrasound, which demonstrated a normal renal and urinary collecting system, a normal aldosterone:renin ratio and an echocardiogram which showed evidence of moderate left concentric hypertrophy. A 24-h urine collection for caetacholamines and metanephrines was carried out and found to be markedly elevated (see Table 1).

**Table 1:** Investigation of urine sample (3×24-h pre-operative urinary caetacholamine and metanephrine collections). Abnormal values are in bold

	**Sample 1**	**Sample 2**	**Sample 3**	**Reference**
Noradrenaline (nmol/24 h)	**7740**	**8660**	**5674**	0–900
Adrenaline (nmol/24 h)	**257**	**350**	**566**	0–230
Dopamine (nmol/24 h)	**1991**	**1990**	**1789**	0–3300
Metanephrine (nmol/24 h)	**3193**	**4500**	**4800**	0–1800
Normetanephrine (nmol/24 h)	28 287	26 400	24 320	0–2800

The patient was then referred to our service for further evaluation. The patient had no past medical history and was not on regular medication. She had been living in Ireland for the past 7 years and was attending a course at a local college. There was no family history of hypertension or endocrine disease. On examination, she had no clinical features to suggest an endocrinopathy and, apart from an elevated blood pressure of 150/100, her clinical examination was entirely normal. A full biochemical profile and endocrine blood panel including neuroendocrine markers were carried out (see Table 2). Radiological localisation with a CT adrenal protocol was carried out and showed a left sided 4.5×3.2 cm adrenal mass with Hounsfield units of 35. A MIBG scan confirmed increased and abnormal uptake by the left-sided adrenal lesion with no other uptake noted. The patient underwent a laparoscopic adrenalectomy after adequate alpha and beta blockade. The patient was re-evaluated 6 weeks after the surgery. She remained normotensive of all medication and repeated urinary caetacholamine and metanephrine collections were within the normal range. Genetic analysis was performed and confirmed a mutation in exon 8 of the *RET* gene.

**Table 2:** Investigation of serum sample

**Serum parameters**	**Results**	**Reference range**
Calcium (mmol/l)	2.33	2.17–2.51
Free T_4_ (pmol/l)	17.10	12–22
Thyroid-stimulating hormone (ng/l)	2.66	0.4–3.8
Parathyroid hormone (ng/l)	32	16–65
Chromogranin A (pmol/l)	25	<60
Chromogranin B (pmol/l)	**180**	<150
Basal calcitonin (ng/l)	**11.3**	0–10.0
Calcitonin 1 min post pentagastrin (ng/l)	**31.3**	0–10.0
Calcitonin 3 min post pentagastrin (ng/l)	**110.0**	0–10.0
Calcitonin 5 min post pentagastrin (ng/l)	**136.0**	0–10.0
Calcitonin 10 min post pentagastrin (ng/l)	**109.5**	0–10.0

**Figure 1:**
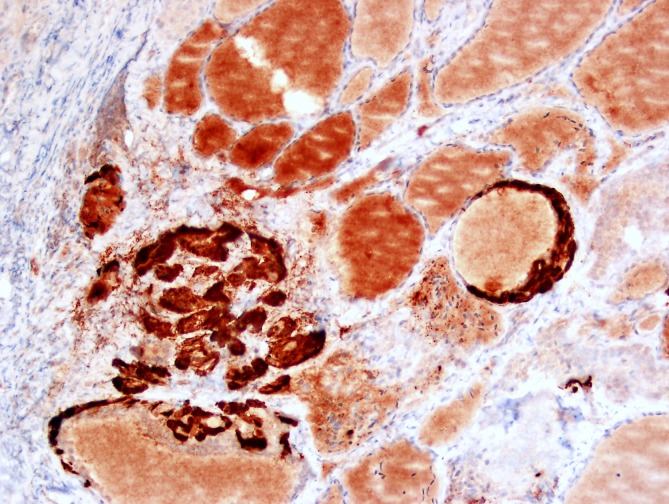
Calcitonin immunohistochemistry highlights large nests of C cells (20×).

Examination of the thyroid gland revealed no clinical abnormality and a pentagastrin stimulation test was carried out. This demonstrated an elevated basal calcitonin and a peak calcitonin of a 156 ng/l post 0.5 μg/kg of pentagastrin by injection (see Table 2). A thyroid ultrasound showed a normal-sized thyroid gland with no radiological abnormalities. The patient underwent a total thyroidectomy and histology, which confirmed the presence of C cell hyperplasia but no focus of medullary thyroid carcinoma (see Fig. 1).

To date, there is no biochemical evidence of primary hyperparathyroidism. We have been unable to provide genetic counselling to first-degree relatives as they are not living in Ireland, but correspondence has been sent to the family regarding the genetic mutation identified. At present genetic analysis is not available to the family, but the mother of the index case has undergone a total thyroidectomy with histology showing evidence of a 0.8×0.8×0.6 cm focus of medullary thyroid carcinoma in the left lobe of the thyroid, with no evidence of extra-thyroidal extension.

## Investigation

For details of the investigation see Tables 1 and 2

### Histology

Histology of the left adrenal gland mass revealed a phaeochromocytoma, composed of nests of polyclonal cells with prominent nucleoli and granular eosinophilic cytoplasm, embedded in a fibrovascular stroma. There was no necrosis, evidence of vascular or capsular invasion and Ki67 proliferation index was <1%.

Thyroid gland showed evidence of C cell hyperplasia with multiple large aggregates (>50 cells) identified bilaterally. There was also evidence of lymphocytic thyroiditis (see Fig. 1).

### Genetic analysis

EDTA blood samples for DNA analysis was collected as per protocol. The genetic analysis was carried out by the method of next generation sequencing at Yorkshire regional DNA laboratory, Leeds, United Kingdom. The following genes were sequenced: *PRKAR1A*, *RET*, *SDHAF2*, *SDHB*, *SDHC*, *SDHD*, *TMEM127*, *VHL* and *MAX*. A heterozygous pathogenic mutation c.1597 G>T (P.Gly533Cys) in exon 8 of the *RET* gene was identified.

## Discussion

In MEN2A, about 90% of adult carriers of the mutated gene will develop MTC, 50% unilateral or bilateral phaeochromocytoma and 20–30% parathyroid tumours. MTC carries the highest morbidity and mortality of the endocrine components of MEN2A. It is well recognised that there is an important genotype–phenotype correlation associated with *RET* gene mutations, which allows risk stratification for at risk family members who harbour *RET* mutations. This risk refers to the age at which MTC presents and the aggressiveness of disease and thus helps plan timely prophylactic thyroidectomy.

Mutations in exon 8 of the *RET* gene have been recognised in association with FMTC since 1999 when Pigney reported on a family where four members had a novel mutation in exon 8 and expressed a FMTC phenotype (1).

It was believed that the risk of developing a phaeochromocytoma in carriers of a mutation in exon 8 of the *RET* gene was low (1). However, evidence has been accumulating that a mutation in exon 8 may be associated with a higher risk of phaeochromocytoma than previously considered. This evidence began in 2007 with the report by Bethanis *et al*. (2), in which he described the first case of a patient who presented with bilateral phaeochromocytoma and was later diagnosed with MTC – first linked to a mutation in exon 8 with MEN2A syndrome. This reported mutation in exon 8 was also a missense mutation (P.Gly533Cys). Since then there have been two other cases of phaeochromocytoma identified in patients with presumed FMTC with mutations in exon 8 (3) (4) and very recently a north American kindred has been described where three family members from three generations were identified with phaeochromocytoma as part of MEN2A syndrome with this same missense mutation (5). Not only is the association with phaeochromocytoma more common with this mutation than previously thought, it can also be the dominant or presenting feature of the disease – as seen in this case.

This is the first reported case of MEN2A syndrome due to a mutation in exon 8 to be reported in Ireland. This patient is originally from Slovenia but there are no reported case reports of MEN2A due to a missense mutation in exon 8 reported from Slovenia at this time. There are two important factors that should be translated into practice as we gain more information about this rare mutation. First, it raises the issue that patients with this mutation labelled as FMTC should undergo lifelong testing to rule out late clinical presentations of phaeochromocytoma and primary hyperparathyroidism as part of MEN2A. Second, genetic analysis for apparently sporadic phaeochromocytoma should include sequencing of exon 8 on the *RET* gene as we have seen that this can be the earliest clinical manifestation of MEN2A with this genotype.

## Patient's perspective

The main symptoms of phaeochromocytoma in my case were high blood pressure, heart palpitations, anxiety, excessive sweating and constant tiredness. These symptoms have gradually worsened throughout my mid- to late-twenties to the point where they were severely affecting my everyday life. Due to my diagnosis and treatment I now have a quality of life I had previously given up on.

## Patient consent

Written informed consent was obtained from the patient for publication of this case report.

## Author contribution statement

R Casey is the first author and contact author and was responsible for the write up of the case. S Prendeville contributed to the pathology write up in the case. C Joyce contributed to the biochemistry and genetics involved in this case. D O'Halloran was the principle investigator and oversaw the write up and management of the case.
